# Draft Genome Sequence of *Aeromonas sobria* Strain 08005, Isolated from Sick *Rana catesbeiana*

**DOI:** 10.1128/genomeA.01352-16

**Published:** 2017-01-12

**Authors:** Qiu-Hua Yang, Chen Zhou, Qi Lin, Zhen Lu, Li-Bin He, Song-lin Guo

**Affiliations:** a Key Laboratory of Cultivation and High-Value Utilization of Marine Organisms in Fujian Province, Fisheries Research Institute of Fujian, Xiamen, China; b Fisheries College, Jimei University, Xiamen, China

## Abstract

*Aeromonas sobria* is a Gram-negative, rod-shaped, and ubiquitous bacterium. We present here the draft genome sequence of *A. sobria* strain 08005, isolated from an infected bullfrog. It is composed of 66 contigs totaling 4,678,951 bp, contains 4,252 coding DNA sequences (CDSs), four rRNAs, and 88 tRNA sequences, and shows the presence of various putative virulence-related genes.

## GENOME ANNOUNCEMENT

*Aeromonas* spp. are rod-shaped, Gram-negative, and facultative anaerobic bacteria ubiquitous in aquatic environments, and they have been implicated in a variety of infections in humans (1–3). Strain 08005 was isolated from infected *Rana catesbeiana* breeding in Xiamen, China, and identified as *A. sobria* Popoff and Véron 1981 by GENIII microstation (Biolog Co., Ltd., USA) with a probability of 93.7%. Although there are no reported human clinical isolates of this species (4), *A. sobria* has been described to be responsible for bacterial pathogens of aquatic animals (5, 6). Here, we report the draft genome sequence of *A. sobria* strain 08005 isolated from infected *Rana catesbeiana*.

Genomic DNA of *A. sobria* strain 08005 was extracted using the genomic purification kit GK1071 (Generay Biotech Co., Ltd., Shanghai, China), according to the manufacturer’s recommended protocol. The quality of the purified genomic DNA was determined using a NanoDrop spectrophotometer (Thermo Scientific) and Qubit 2.0 fluorometer (Life Technologies, Inc.) (7). Whole-genome shotgun sequencing was performed on the Illumina MiSeq system sequencing platform (Illumina, Inc., San Diego, CA, USA). The resulting sequence reads were inspected for data quality using FASTQC version 0.11.3 (Babraham Institute, Cambridge, United Kingdom) (3) and were then filtered using the AdapterRemoval version 1.5.4 (8) and Quake version 0.3 (9) to remove and dynamically trim poor reads. The reads were then assembled *de novo* using Newbler version 2.8 (Roche Diagnostics) and trimmed with GapCloser *in silico*. The draft genome size of *A. sobria* strain 08005 is 4,678,951 bp, with 66 contigs and an average coverage of 109-fold. The overall G+C content of the assembled genome is 57.63%. The *N*_50_ contig size is 168,611 bp.

The draft genome sequence of *A. sobria* strain 08005 was annotated using Glimmer version 3.0 (10). This resulted in the identification of 4,252 predicted coding DNA sequences (CDSs), with an average length of 934.57 bp, four rRNAs (two 5S, one 16S, and one 23S), and 88 tRNA sequences. Various putative genes that could be involved in the pathogenicity mechanism of the bateria were identified, including the immunogenic lipoprotein A (*ilpA*), chemotaxis regulatory protein (*cheY*), proteins related to motility, such as flagellar P-ring protein precursor (*flgI*), flagellar motor switch protein (*fli*), flagellum-specific ATP synthase (*fliI*), flagellar biosynthesis protein (*flhA*), a type VI secretion system, genes associated with drug resistance, such as daptomycin resistance protein (*rpoC*), fluoroquinolone resistance protein (*qnrVC1* and *parC*), colistin resistance protein (*phoP*), and four multidrug resistance efflux pumps. This genomic information will provide a reference genome data set of *Aeromonas*, as well as a useful guide to study the pathogenesis of the organism.

### Accession number(s).

This whole-genome shotgun project of *A. sobria* strain 08005 has been deposited at DDBJ/ENA/GenBank under the accession no. MKFU00000000. The version described in this paper is version MKFU01000000.

## References

[1] Chan KG, Chin PS, Tee KK, Chang CY, Yin WF, Sheng KY 2015 Draft genome sequence of *Aeromonas caviae* strain L12, a quorum-sensing strain isolated from a freshwater lake in Malaysia. Genome Announc 3(2):e00079-15. doi:10.1128/genomeA.00079-15. 25745006 PMC4358393

[2] Moríel B, Cruz LM, Dallagassa CB, Faoro H, de Souza EM, Pedrosa FO, Rego FGM, Picheth G, Fadel-Picheth CMT 2015 Draft genome sequence of *Aeromonas caviae* 8LM, isolated from stool culture of a child with diarrhea. Genome Announc 3(3):e00524-15. doi:10.1128/genomeA.00524-15. 25999559 PMC4440973

[3] Padilla JCA, Bustos P, Castro-Escarpulli G, Sánchez-Varela A, Palma-Martinez I, Arzate-Barbosa P, García-Pérez CA, López-López Mde J, González V, Guo X 2015 Draft genome sequence of *Aeromonas caviae* strain 429865 INP, isolated from a Mexican patient. Genome Announc 3(5):e01240-15. doi:10.1128/genomeA.01240-15. 26494682 PMC4616189

[4] Brenner DJ, Krieg NR, Staley JT 2005 Bergey’s manual of systematic bacteriology, vol 2, part B, 2nd ed. Springer, New York, NY.

[5] Wan-qi C, Pei-fang S 2002 A pathogen study on disease caused by *Aeromonas sobria* in tilapias and drug sensitive test. J Fish Sci China 9:243–246.

[6] Cui-yu B, Ya-bin Z, Pei-jun Z, Lei Z, Dong-ze L, Yue-hong L 2016 Isolation, identification and drug sensitivity test of *Aeromonas sobria* from *Oncorhynchus keta*. Chin J Vet Sci 46:775–780.

[7] Izzati Mohamad NI, Yin WF, Chan KG 2015 Whole-genome sequence of quorum-sensing *Vibrio* *tubiashii* strain T33. Genome Announc 3(1):e01362‑14. doi:10.1128/genomeA.01362-14. 25555738 PMC4293625

[8] Lindgreen S 2012 AdapterRemoval: easy cleaning of next-generation sequencing reads. BMC Res Notes 5:337. doi:10.1186/1756-0500-5-337. 22748135 PMC3532080

[9] Kelley DR, Schatz MC, Salzberg SL 2010 Quake: quality-aware detection and correction of sequencing errors. Genome Biol 11:R116. doi:10.1186/gb-2010-11-11-r116. 21114842 PMC3156955

[10] Delcher AL, Harmon D, Kasif S, White O, Salzberg SL 1999 Improved microbial gene identification with GLIMMER. Nucleic Acids Res 27:4636–4641. doi:10.1093/nar/27.23.4636. 10556321 PMC148753

